# A systematic review of randomised controlled trials on the effectiveness of exercise programs on Lumbo Pelvic Pain among postnatal women

**DOI:** 10.1186/s12884-015-0736-4

**Published:** 2015-11-26

**Authors:** Pei-Ching Tseng, Shuby Puthussery, Yannis Pappas, Meei-Ling Gau

**Affiliations:** Institute for Health Research, University of Bedfordshire, Putteridge Bury, Hitchin Road, Luton LU2 8LE, Bedfordshire, UK; Department of Clinical Education and Leadership & Institute for Health Research, University of Bedfordshire, Putteridge Bury, Hitchin Road, Luton LU2 8LE, Bedfordshire, UK; Graduate Institute of Nurse-Midwifery, National Taipei University of Nursing and Health Sciences, 365, Ming-Te Road, Peitou, Taipei, Taiwan

**Keywords:** Systematic review, Lumbo Pelvic Pain, Exercise, Postnatal women, Randomised controlled trial

## Abstract

**Background:**

A substantial number of women tend to be affected by Lumbo Pelvic Pain (LPP) following child birth. Physical exercise is indicated as a beneficial method to relieve LPP, but individual studies appear to suggest mixed findings about its effectiveness. This systematic review aimed to synthesise evidence from randomised controlled trials on the effectiveness of exercise on LPP among postnatal women to inform policy, practice and future research.

**Methods:**

A systematic review was conducted of all randomised controlled trials published between January 1990 and July 2014, identified through a comprehensive search of following databases: PubMed, PEDro, Embase, Cinahl, Medline, SPORTDiscus, Cochrane Pregnancy and Childbirth Group’s Trials Register, and electronic libraries of authors’institutions. Randomised controlled trials were eligible for inclusion if the intervention comprised of postnatal exercise for women with LPP onset during pregnancy or within 3 months after delivery and the outcome measures included changes in LPP. Selected articles were assessed using the PEDro Scale for methodological quality and findings were synthesised narratively as meta-analysis was found to be inappropriate due to heterogeneity among included studies.

**Results:**

Four randomised controlled trials were included, involving 251 postnatal women. Three trials were rated as of ‘good’ methodological quality. All trials, except one, were at low risk of bias. The trials included physical exercise programs with varying components, differing modes of delivery, follow up times and outcome measures. Intervention in one trial, involving physical therapy with specific stabilising exercises, proved to be effective in reducing LPP intensity. An improvement in gluteal pain on the right side was reported in another trial and a significant difference in pain frequency in another.

**Conclusion:**

Our review indicates that only few randomised controlled trials have evaluated the effectiveness of exercise on LPP among postnatal women. There is also a great amount of variability across existing trials in the components of exercise programs, modes of delivery, follow up times and outcome measures. While there is some evidence to indicate the effectiveness of exercise for relieving LPP, further good quality trials are needed to ascertain the most effective elements of postnatal exercise programs suited for LPP treatment.

## Background

Pain in the lower back and pelvic regions, collectively known as Lumbo Pelvic Pain (LPP), tends to be commonly reported among pregnant and postnatal women with varying prevalence rates. Lumbo Pelvic Pain (LPP) refers to self-reported pain in areas of lower back, anterior pelvis, posterior pelvis, or any combination of these locations [1, 2]. Majority of women report LPP in pregnancy with prevalence rates ranging from 26.5 % to 91 % [3–12]. A substantial number of women continue to experience the pain in the postnatal period with varying intensity and duration [13, 14]. A higher range of variation is reported in LPP prevalence in the postnatal period compared to its prevalence in pregnancy due to apparent differences in follow-up times, methods of measurement and definitions [12, 14–20]. For instance, using a self-rated Visual Analogue Scale (VAS), Líndal et al., [16] reported prevalence rates of 75 % at 3 days after delivery among women who had lower back pain in pregnancy, and 54 % at 90-days after delivery. In a population based survey, Stapleton et al., [18] found that 8 % of women reported the onset of recurrent low back pain soon after pregnancy whereas the figures rose to 13 % at 1 year after child birth. In another prospective cohort study of pregnant women, 28.9 % of all pregnant women had some type of back pain during the index pregnancy and 5 % had pain 3 years after birth [17].

The presence of LPP is often identified and confirmed by diagrammatic representations of self-reported pain location alone or in combination with clinical tests [3, 12, 21–26]. Most LPP is reported in and around the lumbar area, which is responsible for supporting the majority of the upper body weight [27]. Factors associated with LPP occurrence in pregnancy and in the postnatal period include maternal age, parity, high Body Mass Index (BMI), smoking, oral contraceptives, previous history of LPP, uncomfortable working conditions, and lack of exercise [4, 10, 15, 26, 28–31].

Persistent LPP can negatively impact women’s ability to perform daily activities and quality of life. Among postnatal women it has been shown that LPP leads to sleep problems, depression, fatigue, anxiety, and a general inability to doing activities that involve carrying or lifting [25, 32–35]. For instance, Gutke et al., [35] found that women suffering from LPP are three times more likely to experience symptoms of postnatal depression compared to those without. In another study [25], 40 % of women with postnatal LPP reported moderate to severe disability with pain intensity being the major explanatory variable for disability level. The same study also found that the impact of having pelvic girdle pain, combined pain, or lumbar pain were equivalent in terms of disability, pain intensity, health-related quality of life, activity level and kinesiophobia [25].

Different interventions have been used to reduce LPP in general including exercise acupuncture, drugs, therapies using heat/cold, traction, laser, ultrasound, short wave, massage, and corsets [36, 37]. A systematic review of randomised controlled trials of treatment methods to prevent or reduce the incidence or severity of pelvic or back pain in pregnancy have indicated moderate quality evidence suggesting the effectiveness of acupuncture or exercise tailored to the stage of pregnancy, in significantly reducing evening pelvic pain or lumbo-pelvic pain more than usual care alone [38]. The same review also suggested acupuncture as significantly more effective than exercise for reducing evening pelvic pain. Clinical approaches for LPP management have specified the importance of activation of muscles for motor control and stability of the lumbopelvic region [39] and physical exercise has been indicated as a beneficial method to relieve LPP during pregnancy and after child birth [22, 40–42]. Emerging studies on the effectiveness of exercise on LPP among postnatal women, however, appear to indicate mixed findings and do not provide sufficient evidence on their own to inform clinical practice in this area. A systematic synthesis of the existing evidence on the effectiveness of physical exercise on postnatal LPP is yet to be conducted. This review aimed to synthesise findings from randomised controlled trials on the effectiveness of exercise on LPP among postnatal women to inform policy, practice and future research in the area.

## Methods

This review follows the Preferred Reporting Items for Systematic Reviews and Meta Analyses (PRISMA) guidelines (http://www.prisma-statement.org/PRISMAStatement/Checklist.aspx). The review question was framed using Population, Intervention, Comparator, Outcome, and Study design (PICOS) framework. The population comprised of postnatal women who reported LPP onset either in pregnancy or within 3 months after delivery. The interventions comprised of physical therapy with a suite of exercise programs specifically designed to strengthen deep local muscles and global muscles in the lumpopelvic region. The comparators included no therapy; or physical therapy using other methods such as massage relaxation, joint mobilization, manipulation, electrotherapy, hot packs, and simple back strengthening exercises. The primary outcome measure was changes in LPP. This review considered randomised controlled trials published between 1990 and 2014. Randomised controlled trials were eligible for inclusion if the reported intervention comprised of postnatal exercise for women who reported LPP onset during pregnancy or within 3 months after delivery, and the outcome measures included changes in LPP. The review protocol was agreed between the four authors.

A comprehensive search of the following databases was undertaken during February and March 2014 to identify relevant studies: PEDro CINAHL, MEDLINE, PUBMED, SPORTDiscus. Other review data bases such as Cochrane Central Register of Controlled trials, Cochrane Pregnancy and Childbirth Group’s Trials Register, Centre for Reviews and Dissemination University of York, and electronic libraries of authors’ institutions were also searched. Additional sources included Google Scholar and reference list of relevant articles and book chapters. Authors identified through the search process were contacted to identify any further publications. The key search terms used for searches across all databases is provided in Table 1. An updated search was completed in July 2014. The retrieved articles were scanned for relevance based on the title, abstract and full text.

**Table 1:** Key search terms

1	postpartum women OR “postnatal women” OR “after delivery” OR “postpartum period” OR “postpartum females” OR “birth” OR “after birth” OR “natal” OR “perinatal” OR “puerperium”	Population
2	exercise OR “postpartum exercise” OR “postnatal exercise” OR “postpartum training” OR “postpartum practices” OR “abdominal training” OR “exercise prescription” OR “abdominal training “OR “female athlete” OR “physical activities” OR “physical fitness”	Interventions
4	back pain OR “backache” OR “low back pain “OR “lower back pain “OR “upper back pain” OR “high back pain” OR “anterior pelvic pain” OR “posterior pelvic pain” OR “buttocks pain” OR “pelvic pain” OR “symphysis pain” OR “sacroiliac joint pain” OR “pelvic girdle pain” OR “lumbar pelvic pain” OR “lumbosacral pain” OR “lumbar pain” OR “postpartum-related LBP” OR “self management of LBP” OR “vertebrogenic pain”	Outcome
5	core muscles OR “trunk muscles” OR “core stabilisation” OR “transverses abdominis” OR “lumbar multifidus” OR “musculoskeletal” OR “musculoskeletal conditions” OR “musculoskeletal disorders”	
6	physical endurance OR “endurance” OR “core muscle strength”	
7	1 AND 2 AND 3 AND 4 AND 5 AND 6	

### Methodological quality assessment and data analysis

The selected articles were subsequently assessed using the PEDro Scale (http://www.pedro.org.au/english/downloads/pedro-scale/) which consists of 11 items for rating methodological quality of randomised controlled trials. The scale has been used in other reviews [43]. A score of 9–10 on the scale represents ‘excellent’ quality; a score of 6–8 represents ‘good’ quality; and a score of 4–5 represents ‘fair’ quality [43, 44]. Two authors (PcT and SP) independently assessed the risk of bias in all the included trials using Cochrane Collaboration’s tool for assessing risk of bias [45]. The data from selected studies were extracted into tables comprising of study characteristics along with quality assessment ratings for each study as shown in Table 2 and Table 4.

**Table 2:** Summary of study characteristics

Author/Publication year/country	Publication title	Design	Population/Sample	Quality rating
Mens et al., [46] 2000 Netherlands	Diagonal Trunk Muscle Exercises in Peripartum Pelvic Pain: A Randomised Clinical Trial	Randomised controlled trial	Total: 44 women	‘Good’
			Intervention group: 16	
			Control group 1: 14	
			Control group 2: 14	
Stuge et al., [47]^a^ 2004	The Efficacy of a Treatment Program Focusing on Specific Stabilising Exercises for Pelvic Girdle Pain After Pregnancy: A Randomised Controlled Trial	Randomised, single-blind, clinically controlled study with a stratified group design	Total: 81 women	‘Good’
			Intervention group: 40	
			Control group: 41	
			One year postpartum:	
			Intervention group: 39	
Stuge et al., [51]^a^ 2004 Norway	The Efficacy of a Treatment Program Focusing on Specific Stabilising Exercises for Pelvic Girdle Pain After Pregnancy: A Two-Year Follow-up of a Randomised Clinical Trial		Control group: 39	
			Two year postpartum:	
			Intervention group: 30	
			Control group: 35	
Gutke et al., [48] 2010 Sweden	Specific muscle stabilising as home exercises for persistent pelvic girdle pain after pregnancy: a Randomised, Controlled Clinical Trial	Prospective, randomised, single-blinded clinically controlled study	Total: 86 Women	‘Good’
			Intervention group: 32	
			3-month follow-up analysis (*n* = 26)	
			6-month follow-up analysis (*n* = 24)	
			Control group: 54	
			3-month follow-up analysis (*n* = 39)	
			6-month follow-up analysis (*n* = 36)	
Chaudry et al., [49] 2013 Pakistan	Effectiveness of core stabilisation exercises along with postural correction in postpartum back pain	A randomised controlled trial with non-probability sampling	Total: 40 women	‘Fair’
			Intervention group: 20	
			Control group: 20	

^a^Both publications originated from the same trial

## Results

### Study selection

**Fig. 1:**
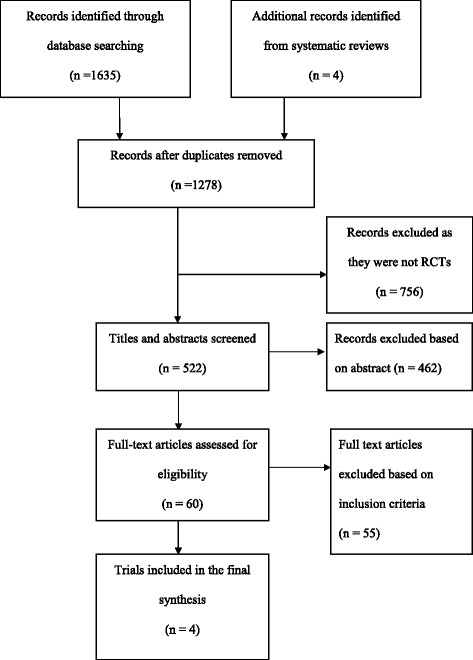
Flowchart of study selection process

The results of the search and study selection are shown in Fig. 1. The search process produced 1639 titles and 1278 records were retrieved after duplicates were removed. 756 were further excluded as these were not randomised controlled trials. The remaining 522 were exported to the reference software Mendeley and the titles and abstracts were screened. 60 articles were selected for full text screening. Following full text screening, 55 articles were excluded due to discordance with the inclusion criteria. Finally, five articles originating from four trials were included in the review as shown in Table 2.

The methodological quality assessment using the PEDro scale revealed a mean score of 6 (range = 4–8). Three trials [46–48] were found to be of ‘good’ methodological quality. Although one trial [49] was rated as of ‘fair’ methodological quality, the reported information was inadequate to make a full assessment. This trial was included in the review based on the appropriateness of the study design and reported outcome measures. The results of the risk of bias assessment using the Cochrane tool are presented in Table 3. All the studies except one [49] were at low risk of bias on key domains such as sequence generation, allocation concealment, blinding of participants and personnel, completeness of outcome data for each main outcome, and selective reporting. In Chaudry et al’s trial [49], patients were reported to be randomly allocated, but the available information was largely insufficent to make clear judgement on other domains.

**Table 3:** Risk of bias

Domains	Risk of bias			
	Mens et al. [46]	Stuge et al. [47]^a^ & [51]^a^	Gutke et al. [48]	Chaudry et al. [49]
Random sequence generation	Low risk of bias	Low risk of bias	Low risk of bias	Low risk of bias
Allocation concealment	Low risk of bias	Low risk of bias	Low risk of bias	Unclear risk of bias
Blinding of participants and personnel	Unclear risk of bias	Low risk of bias	Low risk of bias	Unclear risk of bias
Incomplete outcome data	Low risk of bias	Low risk of bias	Low risk of bias	Unclear risk of bias
Selective reporting	Low risk of bias	Low risk of bias	Low risk of bias	Unclear risk of bias
Other sources of bias	Low risk of bias	Low risk of bias	Low risk of bias	Unclear risk of bias

^a^Both publications originated from the same trial

Although the interventions included exercise programs, the components of the intervention, outcome measures, and follow up times were too diverse to allow a meta-analysis of the study findings. This was further confirmed by testing homogeneity with the Meta-Analysis Add-In for Microsoft Excel software package [50] and hence a narrative synthesis was undertaken.

### Study characteristics

The study participants included 251 postnatal women reported in four trials who experienced LPP onset either in pregnancy or within 3 months after child birth as described in Table 2. The overall sample size in individual trials ranged from 40 to 86 [48, 49] with the size of the intervention group varying between 16 and 41 women [46, 51]. Two articles had originated from the same trial reporting outcome measures at different intervals such as the week after the 18–20 week intervention, 1-year, and 2-years postpartum [47, 51]. One study divided participants into three groups: an experimental group with 16 postnatal women and two control groups with 14 each [46]. The included trials were from different countries: Netherlands [46], Norway [47, 51], Sweden [48] and Pakistan [49].

The assessment data were collected at different time points such as baseline [46–49, 51]; soon after intervention [46]; at 3- and 6- months follow up [48]; and at 1- and 2- years after delivery [47, 51]. Only one trial assessed the long term effect of the intervention with outcomes reported at 1- and 2 -year follow-up periods [47, 51]. One trial did not clearly report the time points when the assessment data was collected [49].

### Interventions and comparators

The interventions consisted of various exercise programs as presented in Table 2. Three trials used stabilisation exercise programs as the intervention - either specific [48] or core [47, 49, 51], while the third trial used diagonal trunk muscle systems training program [46]. The core stabilisation exercise program used by Stuge et al., [47, 51] was focused on training the deep local muscles (the transverse abdominal wall muscles with co-activation of the lumbar multifidus in the lumbosacral region) and global muscles (m. gluteus maximus, m. latissimus dorsi, the oblique abdominal muscles, m. erector spinae, m. quadratus lumborum, and hip adductors and abductors). The initial focus of this exercise was on specific contraction of the transverse abdominal muscles. In addtion to stabilisation exercises, postural correction techniques in different positions such as supine, crook lying, half sitting and prone were also employed for the intervention group in another trial [49]. The specific stabilising exercises reported by Gutke et al., [48] focused on strengthening the transversely oriented abdominal, lumbar multifidus, and the pelvic floor muscles, and on improving motor control and stability.

The interventions were varied in their frequency and duration. The frequency of the exercise ranged from ≥ 2 times per day [48] to three days per week [46, 47, 51]. The exact frequency of the exercise was not reported in one trial [49]. In this trial, women in the treatment group were were given three exercise sessions of half an hour during their stay in the hospital after birth. After discharge from the hospital, these women were called back for follow up sessions of 30 to 40 min treatment [49]. The total reported duration of the intervention was between 8 weeks [46] and 20 weeks [47, 51] although this information was not available in the case of two trials [48, 49]. Co-interventions such as the use of a pelvic belt and pain medication were reported to be used for the experimental and control group in one trial [46]. The methods of delivering the interventions differed across trials and included a videotape with instruction of exercises to be performed at home without supervision [46]; individualized exercise program performed mainly at home with guidance by the physical therapist with adjustments performed once a week or fortnightly [47, 51]; home training with individual guidance and adjustment of the exercise program every two weeks by one of two treating physiotherapists [48]; and treatment sessions at the hospital [49]. Compliance was measured using a training diary in two trials [47, 48, 51] and a designated form in another one [46]. This information was not available in one trial [49]. The home-based approach in one trial was reported to be a barrier to control for compliance with diaries not handed in as expected [48]. One trial reported high compliance with the treatment [47, 51]. Compliance was less optimal in two trials [46, 48]. In one trial, 25 % of the subjects in the experimental group stopped their exercise programme before the end of the study because of increase in pain [46] and only 78 % of the women in the treatment group reached stage 3 of the treatment programme in the other [48]. No compliance information was reported in one trial [49].

The comparators included longitudinal trunk muscle systems training [46]; physical therapy using ergonomics massage, joint mobilization, manipulation, electrotherapy, hot packs [47,51]; simple back strengthening exercises [49]; or no exercise [46, 48]. Mens et al. [46] included two comparison groups - one group with instructions to train the longitudinal trunk muscle system involving rectus abdominis muscle, longitudinal parts of the erector spinae muscle, and quadratus lumborum muscle, and the other instructed to refrain from exercise.

### Outcomes

#### Primary outcome: Changes in LPP

The outcomes reported in the trials are presented in Table 4. The primary outcome considered for this review is the change in LPP intensity among postnatal women at different follow up intervals. All the included trials reported changes in pain intensity and related variables as an outcome measure based on assessment using the Visual Analogue Scale (VAS) which is a uni-dimensional measure of pain intensity [52]. Pain intensity was assessed in the morning and in the evening in two trials as a primary outcome measure [46, 47, 51]; whereas current pain and average pain during the previous week was used as the measure in another trial [48]. The latter trial also assessed pain frequency (always day and night to several times per week, or occasionally to never) [48]. Although VAS was reported to be used to measure pain intensity, the actual changes in pain intensity was unclear in one trial [49]. Gluteal pain provoked by the Posterior Pelvic Pain Provocation (PPPP) test on the left and right sides was also reported as secondary outcome in one trial [46].

**Table 4:** Summary of findings

Author/Publication year	Intervention	Comparator	Intervention duration and frequency	Outcome measures	Effectiveness of the intervention (*P*<05)
Mens et al., [46] 2000	Instructions given by videotape with training of the diagonal trunk muscles (*n*=16).	Comparator 1: Instructions given by videotape with training of the longitudinal trunk muscles (*n*=14).	8-week duration.	Intensity of pain and fatigue in the morning and evening based on Visual Analogue Scale (VAS).	No significant differences in pain intensity, fatigue, HQRL, or mobility measures between the experimental group and both control groups.
			Light exercises to be performed 3 times per day and heavy exercises 3 times per week		
				Health-related quality of life (HQRL) based on Nottingham Health Profile (NHP).	
		Comparator 2: Instructions given by videotape without exercises (*n*=14).		Gluteal pain provoked by the Posterior Pelvic Pain Provocation (PPPP) test on the left and right sides.	Experimental group scored better than the control groups with repect to gluteal pain provoked by the PPPP test on the right side.
				Mobility of pubic symphysis (radiographic examination).	
Stuge et al., [47]^a^ 2004 & Stuge et al., [51]^a^ 2004	Physical therapy with specific stabilising exercises (*n*=40).	Physical therapy without specific stabilising exercises (*n*=41).	18 to 20 weeks duration.	Pain intensity in the morning and evening based on VAS.	After the intervention and 1 year follow up:
				Functional status (Oswestry LBP Disability Questionnaire). Health-related quality of life (SF-36 Health survey).	Pain intensity in the morning and evening was significantly reduced in the intervention group. Functional status in the intervention group significantly better than the control group.
				Physical endurance (Sӧrensen Test, ASLR test).	
					Health-related quality of life shows significant improvement in the intervention group with largest effect in physical function, role physical and bodily pain.
			3 days a week with a daily duration of 30 to 60 min		
					Significant differences in functional status, evening pain, and morning pain between the groups were maintained 2 years after delivery.
					Health-Related Quality of Life at 2 years after delivery revealed that significant differences persisted between the groups in physical functioning, role physical, and bodily pain.
					No significant differences between the 2 groups were seen for the other 5 subscales (general health, vitality, social functioning, role emotional, and mental health).
Gutke et al., [48] 2010	Specific stabilising exercises focused on the transversely Oriented abdominal, the lumbar multifidus, and the pelvic floor muscles.	No exercise.	Total duration not reported ≥ 2 times per day and to perform each exercise with 10 repetitions.	Disability based on the Oswestry Disability Index (ODI) version 2.0.	For ODI, no difference could be demonstrated between the intervention and control groups at 3- or 6-month follow-up. Significant difference in pain frequency was demonstrated between the two groups at the 3-month follow-up in favour of the intervention group.
		Instructed to resume normal activities.			
				Pain intensity measured with VAS (0–100 mm) for current pain and average pain during the previous week.	
				Pain frequency (always, day and night to several times per week, or occasionally to never).	
				Health related quality of life (HRQL) measured using EuroQol instrument (EQ-5D and EQ-VAS).	
					No differences could be found between the groups regarding pain intensity,
				Wellbeing measured with VAS (0–100 mm) with defined end-points (low value indicating high wellbeing).	HRQL or wellbeing.
Chaudry et al., [49] 2013	Core stabilisation exercises along with postural correction in different positions.	Simple back strengthening exercises in different positions.	Total duration not reported.	Back pain (Visual analogue scale VAS).	Significant improvement in ADLs and IADLs in intervention group compared to control group.
			3 sessions of half an hour during the stay in hospital.	Activities of Daily Livings (ADLs) and Instrumental Activities of Daily Livings (IADLs)	
					Significant improvement in muscle power in intervention group compared to control group.
				Mobility (dependent and independent).	
				Muscles power. Manual Muscle Testing (MMT).	
				Pedal edema.	Significant improvement in mobility in intervention group compared to control group.
					Intervention group showed improvement in edema compared to control group, but p-value was insignificant.

^a^Both publications originated from the same trial

In terms of the effectiveness of the exercise program on LPP, one trial [47, 51] reported significant positive effect on pelvic pain intensity as a result of the exercise. This trial found significant reductions in pain intensity in the morning and evening during the intervention period and at 1- and 2-year follow-ups, with a better reduction of pelvic girdle pain in the intervention group compared to the control group [47, 51]. The authors observed biggest improvements in pain intensity during the intervention period of 20 weeks, with a further but slow improvement over the 6 months following treatment, which was also maintained 2 years after delivery. However, low levels of pain sustained in the intervention group 2 years after delivery [47, 51]. Although another trial reported significant improvements in back pain related variables such as restriction in Activities of Daily Livings (ADLs), and Instrumental Activities of Daily Livings (IADLs), changes in pain intensity was not reported as such [49]. Gutke et al., [48] reported significant difference in pain frequency between the intervention and control groups at 3-month follow-up in favour of the intervention group, but did not find any differences between the groups with respect to pain intensity, or other related variables such as health related quality of life (HRQDL) and wellbeing. Mens et al., [46] did not find any significant difference with respect to the severity of pain in the morning and evening or related fatigue between the experimental group and both control groups. However, the intervention group scored better than the control groups with respect to changes in the gluteal pain provoked by the PPPP test scores on the right side [46]. Within-group comparisons in three trials showed a decrease in LPP intensity and associated variables in both experimental and control groups at different follow-up intervals compared to baseline [46–48, 51].

#### Other LPP related outcomes

A number of other LPP related outcome measures were also reported as shown in Table 4. Two trials reported changes in functional status or disability measured using Oswestry Lower Back Pain Disability Questionnaire (ODI) [47, 48, 51] and Disability Rating Index (DRI) [51]. Stuge et al. [47, 51] reported significant improvements in functional status in the intervention group compared to the control group at one week after the intervention and at 1-and 2-year follow ups. However, Gutke et al., [48] could not find any difference with respect to functional status between the 2 groups at 3- or 6-month follow-ups.

Changes in health related quality of life in the intervention and control groups were reported in three trials using instruments such as SF-36 Health Survey [47, 51], EuroQol instrument (EQ-5D and EQ-VAS) [48], and Nottingham Health Profile (NHP) [46]. Using SF-36 Health Survey, Stuge et al., [47, 51] assessed health related quality of life at the time of entry, within one week after intervention, 1- and 2-years postnatal. On health related quality of life measurements, the same trial reported significant differences between the experimental and control groups in physical functioning, role physical, and body pain following the intervention and at 1- and 2-years after delivery [47, 51]. Using NHP, Mens et al., [46] reported overall improvement among study participants on NHP pain scale at 8 weeks of intervention compared to baseline, but could not find any statistically significant difference between the intervention and control groups. No differences were detected between the groups by Gutke et al., [48] on EuroQol instrument (EQ-5D and EQ-VAS) on health related quality of life or wellbeing measured with VAS with defined end-points (low value indicating high wellbeing).

Changes in physical mobility was reported by Mens et al. [46] using radiographic examination to assess mobility of the pubic symphysis on left and right lower extremities at 8 weeks after the intervention [46]. Although there was an overall improvement in physical mobility among participants at 8 weeks of intervention compared to baseline, there was no statistically significant difference between the experimental and comparison groups [46]. Another study reported marked improvement in mobility dependence among the experimental group compared to control group after following core stabilisation exercises and postural correction [49].

Changes in physical endurance was reported based on physical examinations and tests such as Sorensen Test and Active Straight Leg Raising (ASLR) test [47] and muscle function test [48]. Stuge et al., [47] used Sorensen Test and ASLR test at the time of entry, within one week after intervention and one year after delivery and found improvements in physical endurance with statistically significant differences between the groups in favour of the intervention. Gutke et al., [48] found significant difference between the intervention and control groups for the mean hip extension remaining at 3-month follow up. Within-group comparisons in the same study also showed improvements in both groups in several global muscles, but not in the pelvic floor muscles at 3- and 6-months follow up compared to baseline [48].

## Discussion

The current review was undertaken to synthesise the evidence from randomised controlled trials on the effectiveness of physical exercise on LPP among postnatal women. Despite a comprehensive search, the authors did not find any other systematic reviews focusing on the effectiveness of exercise on LPP among women after child birth. Our review indicates that only a small number of randomised controlled trials have evaluated the effectiveness of exercise on LPP among postnatal women either as a primary or secondary outcome. Further, existing trials appear to suggest inconsistent findings and do not adequately allow estimates of effect in either direction. Among the four trials included in our review, involving 251 post natal women, three were rated as of ‘good’ methodological quality, with a score of 6–8 on a 10 point assessment scale, indicating fairly good methodological rigor. Among these, one trial that involved physical therapy with specific stabilising exercises proved to be effective in terms of reducing LPP intensity both after the intervention and at 1- and 2- year follow ups [47, 51]. The same trial also showed significant positive effect of the exercise program on other related variables such as functional status, health related quality of life and physical endurance [47, 51]. The remaining two trials that were rated as of ‘good’ quality did not show any beneficial impact with respect to LPP intensity [46, 48]. However, improvements in gluteal pain on the right side was found in the intervention group in one trial [46], and a significant difference in pain frequency between the two groups at 3-month follow-up in the other [48]. Reportedly, many participants in the treatment group in one trial complained of increasing pain during the exercises with the majority attributing the pain to the exercise aimed at strengthening the hip extensors [46].

The inconsistent findings found in our review may be attributed to methodological factors, variability in the intervention elements and the way the intervention was administered. Previous research has highlighted the importance of activation of muscles for motor control and stability of the lumbo pelvic region [39, 53], and a recent pre and post experimental study using convenient sampling has suggested lumbo-pelvic stabilisation exercises to be beneficial for improving trunk muscle endurance, pain and functional ability in women with postnatal lumbo-pelvic pain [54]. Among the trials included in our review, only one included thoroughly instructed regularly supervised high quality exercises designed to involve all relevant muscles of the pelvic girdle [47, 51]. There were also marked differences across trials with respect to type of exercises, frequency and duration, and the way the exercises and instructions were administered. Compliance to the intervention is also likely to significantly influence the outcomes and is an important indicator of an intervention’s feasibility for future implementation. Among the trials included in our review, only one trial reported good compliance [47, 51]. The ability to exercise without provoking pain, possibility of training at home under the guidance of a therapist, use of a training diary, the ability to gradually increase the resistance of individually adapted exercises and the integration of muscle control into functional tasks were all found to be important to encourage compliance [47, 51]. Although VAS was used as measure of pain intensity in all the trials, a range of pain related outcome measures were reported across trials. As evident in our review and as has been indicated by other researchers, a standardised set of outcome measures to accurately capture LPP is yet to be developed [46].

The rigorous methodological approach based on a well-defined research question with a comprehensive search strategy, clear inclusion and exclusion criteria, standardised quality assessment techniques, and structured data extraction make our findings valid and reliable. The review has certain limitations, however. Although the narrative synthesis allowed for a thorough discussion on the effectiveness of the interventions, a meta-analysis was not feasible for making estimates of strength of effect due to variations in intervention components, outcome measures, follow up times and study quality among the selected studies. The restriction to randomised controlled trials as inclusion criteria might have resulted in the exclusion of non-randomised and other experimental studies that have yielded useful findings. However, randomised controlled trials are deemed to be the most rigorous method to determine the presence of a cause-effect relationship between an intervention and outcome, and therefore the highest quality of evidence for a systematic review [55, 56]. In spite of a comprehensive search, we were able to identify only four randomised controlled trials that met our inclusion criteria. However, three of the four selected trials were of fairly good methodological quality with blinded assessments and standardised and validated data collection tools to ensure internal validity and the robustness of the findings. Although the authors of one trial [49] were contacted for additional information about methodological aspects, this information could not be obtained. We were also unable to explore any potential publication bias resulting from exclusion of unpublished randomised controlled trials or findings reported in grey literature.

## Conclusion

Although postnatal exercise is routinely recommended to women, our review indicates a paucity of methodologically rigorous research studies to make reliable conclusions with respect to the effectiveness of physical exercise on LPP amongst postnatal women. An individually tailored program reported in a fairly good quality trial, with stabilising exercises involving all relevant muscles, delivered under the guidance of a therapist with high treatment compliance was shown to be effective on LPP and other related variables. Further high quality randomised trials with controlled co-interventions and standardised outcome measures are needed to identify the most effective combination of exercise elements that can have an effect on reducing LPP and the associated health and well-being factors.

As a substantial number of women tend to be affected by LPP following pregnancy and birth with significant potential implications for the women, their families, and the society as a whole, effectively managing LPP is an issue for all stakeholders concerned with maternal and women’s health. While physical therapy involving exercise programs tends to be one of the treatment approaches used to relieve LPP, ascertaining its effectiveness is a matter of importance to policy, practice and research in the area.

## Abbreviations

ASLR: Active straight leg raising
BMI: Body mass index
LPP: Lumbo pelvic pain
NHP: Nottingham Health Profile
VAS: Visual analogue scale
